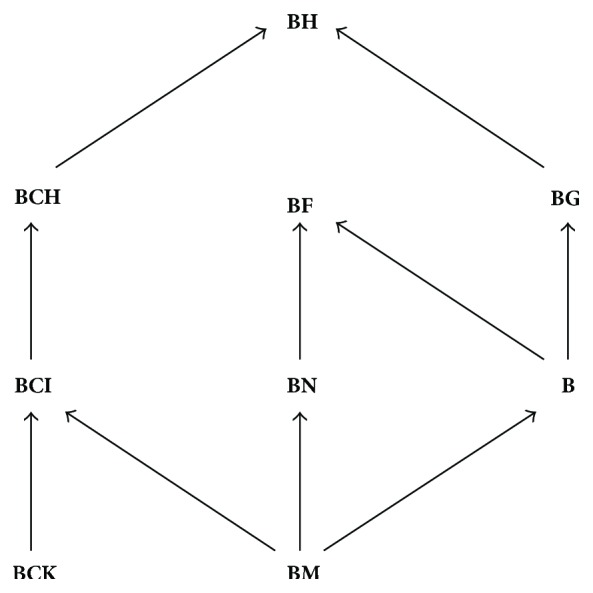# (Fuzzy) Ideals of BN-Algebras

**DOI:** 10.1155/2015/925040

**Published:** 2015-06-01

**Authors:** Grzegorz Dymek, Andrzej Walendziak

**Affiliations:** ^1^Institute of Mathematics and Computer Science, The John Paul II Catholic University of Lublin, Konstantynów 1H, 20-708 Lublin, Poland; ^2^Institute of Mathematics and Physics, Siedlce University, 3 Maja 54, 08-110 Siedlce, Poland

## Abstract

The notions of an ideal and a fuzzy ideal in BN-algebras are introduced. The properties and characterizations of them are investigated. The concepts of normal ideals and normal congruences of a BN-algebra are also studied, the properties of them are displayed, and a one-to-one correspondence between them is presented. Conditions for a fuzzy set to be a fuzzy ideal are given. The relationships between ideals and fuzzy ideals of a BN-algebra are established. The homomorphic properties of fuzzy ideals of a BN-algebra are provided. Finally, characterizations of Noetherian BN-algebras and Artinian BN-algebras via fuzzy ideals are obtained.

## 1. Introduction

In 1966, Imai and Iséki [1] introduced the notion of a BCK-algebra. There exist several generalizations of BCK-algebras, such as BCI-algebras [2], BCH-algebras [3], BCC-algebras [4], BH-algebras [5], and d-algebras [6]. Neggers et al. defined B/BM/BG-algebras [7–9] and showed that the class of all B-algebras is a proper subclass of the class of all BG-algebras. They also proved that an algebra is a BM-algebra if and only if it is a 0-commutative B-algebra (therefore, every BM-algebra is a B-algebra). In [10], it is shown that the class of 0-commutative B-algebras is the class of *p*-semisimple BCI-algebras (and hence any BM-algebra is a BCI-algebra). The class of BM-algebras contains Coxeter algebras (see [8]). Some other connections between BM-algebras and its related topics are studied in [11]. Walendziak introduced in [12] the concept of BF-algebras, which is a generalization of B-algebras and BN-algebras defined by C. B. Kim and H. S. Kim [13]. An interesting result of [13] states that an algebra is a BN-algebra if and only if it is a 0-commutative BF-algebra.

We will denote by **B** **C** **K** (resp., **B** **C** **I**/**B** **C** **H**/**B** **H**/**B**/**B** **M**/**B** **G**/**B** **F**/**B** **N**) the class of all BCK-algebras (resp., BCI/BCH/BH/B/BM/BG/BF/BN-algebras). The interrelationships between some classes of algebras mentioned before are visualized in Figure 1 (an arrow indicates proper inclusion; that is, if **X** and **Y** are classes of algebras, then **X** → **Y** means **X** ⊂ **Y**).

In this paper we consider ideals and fuzzy ideals in BN-algebras. In Section 2, similarly to BCK/BCI/BCH/BH/BF-algebras (see [5, 12, 14–16]), we define the concept of an ideal. We also introduce the notions of normal ideals and normal congruences. We investigate the properties of them and prove that there is a one-to-one correspondence between normal ideals and normal congruences of a BN-algebra. Moreover we obtain the isomorphism theorem for BN-algebras. In this section we also give all what is necessary, to make the paper self-contained. In Section 3 we define fuzzy ideals in BN-algebras (fuzzy ideals of BCK/BCI/BCC/BF-algebras are considered in [17–20]) and provide conditions for a fuzzy set to be a fuzzy ideal. Given a fuzzy set *μ*, we make the least fuzzy ideal containing *μ*. This leads us to show that the set of fuzzy ideals of a BN-algebra is a complete lattice. Moreover, the homomorphic properties of fuzzy ideals are provided. Finally, characterizations of Noetherian BN-algebras and Artinian BN-algebras in terms of fuzzy ideals are given in Section 4. Noetherian BCK/BCI/BM/BF-algebras are studied in [21–26].

## 2. On BN-Algebras: Ideals

An algebra (*A*; ∗, 0) of type (2,0) is called a* Coxeter algebra* [27] if for all *x*, *y*, *z* ∈ *A* the following identities hold:

(B1) *x*∗*x* = 0,
(B2) *x*∗0 = *x*,
(C) (*x*∗*y*)∗*z* = *x*∗(*y*∗*z*).

**Remark 1 .**

Kim et al. [27] showed that a Coxeter algebra is equivalent to an abelian group all of whose elements have order 2. Therefore, if (*A*; ∗, 0) is a Coxeter algebra, then the operation ∗ is commutative and associative.

We say that (*A*; ∗, 0) is a* BM-algebra* [8] if it satisfies (B2) and

(BM) (*z*∗*x*)∗(*z*∗*y*) = *y*∗*x*.

**Proposition 2 (see [8]).**

If (*A*; ∗, 0) is a BM-algebra, then, for any *x*, *y*, *z* ∈ *A*,

- 0∗(*x*∗*y*) = *y*∗*x*,
- (*x*∗*y*)∗*z* = (*x*∗*z*)∗*y*.

**Proposition 3 .**

If (*A*; ∗, 0) is a BM-algebra, then, for all *x*, *y* ∈ *A*,

- *x*∗(*x*∗*y*) = *y*,
- if *x*∗*y* = 0, then *x* = *y*.

**Proof**

(i) Substituting *z* = *x* and *x* = 0 in (BM), we have (*x*∗0)∗(*x*∗*y*) = *y*∗0. Applying (B2) we get (i).

(ii) follows from (i) and (B2).

An algebra (*A*; ∗, 0) of type (2,0) is called a* BF-algebra* [12] if it satisfies (B1), (B2), and

(BF) 0∗(*x*∗*y*) = *y*∗*x*.

**Definition 4 .**

An algebra (*A*; ∗, 0) of type (2,0) is called a* BN-algebra* [13] if (B1), (B2), and

(BN) (*x*∗*y*)∗*z* = (0∗*z*)∗(*y*∗*x*)

hold for all *x*, *y*, *z* ∈ *A*.

Let (*A*; ∗, 0) be a BN-algebra. We define a binary relation ≤ on *A* by *x* ≤ *y* if and only if *x*∗*y* = 0. It is easy to see that, for any *x* ∈ *A*, if *x* ≤ 0, then *x* = 0.

**Example 5 .**

Let *ℝ* be the set of real numbers and let *ℛ* = (*ℝ*; ∗, 0) be the algebra with the operation ∗ defined by

(1) $$\begin{array}{c} x*y=\left\{\begin{array}{cc} x, & \text{if}\;\;y=0,\\ y, & \text{if}\;\;x=0,\\ 0, & \text{otherwise}. \end{array}\right. \end{array}$$

Then *ℛ* is a BN-algebra.

**Example 6 (see [13]).**

Let *A* = {0,1, 2,3} and ∗ be defined by the following:

(2) ∗012300123110112210133110

Then (*A*; ∗, 0) is a BN-algebra.

**Example 7 .**

Let (*G*; +, 0) be an abelian group. If we define *x*∗*y* = *x* − *y*, for all *x*, *y* ∈ *G*, then, by Theorem 2.15 of [13], (*G*; ∗, 0) is a BN-algebra.

**Proposition 8 (see [13]).**

If (*A*; ∗, 0) is a BN-algebra, then, for all *x*, *y* ∈ *A*,

- 0∗(0∗*x*) = *x*,
- 0∗(*x*∗*y*) = *y*∗*x*,
- *y*∗*x* = (0∗*x*)∗(0∗*y*),
- *x*∗*y* = 0⇒*y*∗*x* = 0,
- 0∗*x* = 0∗*y*⇒*x* = *y*.

From [13] we obtain the following relation: **B** **M** ⊂ **B** **N** ⊂ **B** **F**.

From now on, *𝒜* always denotes a BN-algebra (*A*; ∗, 0). We introduce the notion of an ideal in BN-algebras.

**Definition 9 .**

A subset *I* of *A* is called an* ideal* of *𝒜* if it satisfies

$\left({\text{I}}_{1}\right)$ 0 ∈ *I*,
$\left({\text{I}}_{2}\right)$ *x*∗*y* ∈ *I* and *y* ∈ *I* imply *x* ∈ *I* for any *x*, *y* ∈ *A*.

We will denote by Id(*𝒜*) the set of all ideals of a BN-algebra *𝒜*.

**Example 10 .**

Let *ℛ* = (*ℝ*; ∗, 0) be the BN-algebra given in Example 5. Observe that Id(*ℛ*) = {{0}, *ℝ*}. Indeed, let *I* be an ideal of *ℛ* and suppose that *I* ≠ {0}. Then *a* ∈ *I* for some *a* ∈ *ℝ* − {0}. Let *b* ∈ *ℝ*. Obviously, *b*∗*a* ∈ *I* and hence *b* ∈ *I*. Therefore, *I* = *ℝ*.

**Proposition 11 .**

Let *I* ∈ Id (*𝒜*) and *x*, *y* ∈ *A*. If *x* ≤ *y* and *y* ∈ *I*, then *x* ∈ *I*.

**Proof**

Let *x* ≤ *y* and *y* ∈ *I*. Then *x*∗*y* = 0 ∈ *I* and *y* ∈ *I*. Hence *x* ∈ *I*.

A nonempty subset *S* of *A* is called a* subalgebra* of *𝒜* if *x*∗*y* ∈ *S* for any *x*, *y* ∈ *S*. It is easy to see that if *S* is a subalgebra of *𝒜*, then 0 ∈ *S*.

**Example 12 .**

Let *𝒵* = (*ℤ*; −, 0), where *ℤ* is the set of all integers. We know that *𝒵* is a BN-algebra (see Example 7). Obviously, a nonempty subset *S*⊆*ℤ* is a subalgebra of *𝒵* if and only if *a* − *b* ∈ *S* for all *a*, *b* ∈ *S*; that is, *S* is a subgroup of the group of integers. Therefore,

(3) ∅S⊆Z  is a subalgebra of  Z⇔S=mk:k∈Z  for some  m∈N∪0.

**Remark 13 .**

Consider the BN-algebra *𝒜* = (*A*; ∗, 0) given in Example 6. We have Id(*𝒜*) = {{0}, {0,2}, {0,3}, {0,2, 3}, *A*}. Observe that the ideal *I* = {0,2, 3} is not a subalgebra of *𝒜* and *S* = {0,1} is a subalgebra of *𝒜* but it is not an ideal.

**Remark 14 .**

It is easy to prove that the intersection of an arbitrary number of ideals of a BN-algebra *𝒜* is an ideal of *𝒜*. It is also not hard to show that the union of an ascending sequence of ideals of *𝒜* is an ideal of *𝒜*.

A nonempty subset *N* of *A* is said to be* normal* in *𝒜* if

(4) $$\begin{array}{c} \left(x*a\right)*\left(y*b\right)\in N \end{array}$$

for any *x*∗*y*, *a*∗*b* ∈ *N*. We say that an ideal *I* of *𝒜* (resp., a subalgebra *S* of *𝒜*) is* normal* if the set *I* (resp., the set *S*) is normal. We will denote by Id_*N*_(*𝒜*) the set of all normal ideals of *𝒜*.

**Remark 15 .**

It is easy to see that {0}, *A* ∈ Id(*𝒜*). The ideal *A* is normal, but {0} is not normal in general (see the example below).

**Example 16 .**

Consider the set *A* = {0,1, 2,3} with the operation ∗ defined by the following:

(5) ∗012300123110302230233020

It is easily seen that (*A*; ∗, 0) is a BN-algebra. The ideal *I* = {0} is not normal because 1∗3 = 0 ∈ *I*, but (2∗1)∗(2∗3) = 3∗2 = 2 ∉ *I*.

**Proposition 17 .**

If *I* is a normal ideal of *𝒜*, then *I* is a subalgebra of *𝒜*.

**Proof**

Let *x*, *y* ∈ *I*. Since *I* is normal, we conclude that (*x*∗*y*)∗(*x*∗0) ∈ *I*; that is, (*x*∗*y*)∗*x* ∈ *I*. From the definition of an ideal we have *x*∗*y* ∈ *I*. Thus *I* is a subalgebra of *𝒜*.

**Remark 18 .**

The BN-algebra (*A*; ∗, 0) given in Example 16 shows that the converse of Proposition 17 does not hold. Indeed, {0} is a subalgebra of *𝒜* but it is not a normal ideal.

**Proposition 19 .**

Every ideal of a Coxeter algebra is normal.

**Proof**

Let *𝒜* = (*A*; ∗, 0) be a Coxeter algebra and let *I* be an ideal of *𝒜*. Let *x*, *y*, *a*, *b* ∈ *A* and suppose that *x*∗*y*, *a*∗*b* ∈ *I*. Since *I* is a subalgebra, we have (*x*∗*y*)∗(*a*∗*b*) ∈ *I*. From Remark 1 it follows that (*x*∗*a*)∗(*y*∗*b*) ∈ *I*. Thus *I* is normal.

**Lemma 20 .**

Let *I* be a normal ideal of a BN-algebra *𝒜* and *x*, *y* ∈ *A*. Then

- *x* ∈ *I*⇒0∗*x* ∈ *I*,
- *x*∗*y* ∈ *I*⇒*y*∗*x* ∈ *I*.

**Proof**

(a) Let *x* ∈ *I*. Then *x* = *x*∗0 ∈ *I*. Since *I* is normal, (0∗*x*)∗(0∗0) ∈ *I*. Hence, 0∗*x* ∈ *I*.

(b) Let *x*∗*y* ∈ *I*. By (a), 0∗(*x*∗*y*) ∈ *I*. Applying Proposition 8(ii) we have *y*∗*x* ∈ *I*.

**Proposition 21 .**

Let *𝒜* be a BN-algebra and let *S*⊆*A*. Then *S* is a normal subalgebra of *𝒜* if and only if *S* is a normal ideal.

**Proof**

Let *S* be a normal subalgebra of *𝒜*. Clearly, 0 ∈ *S*. Suppose that *x*∗*y* ∈ *S* and *y* ∈ *S*. Then 0∗*y* ∈ *S*. Since *S* is normal, we have (*x*∗0)∗(*y*∗*y*) ∈ *S*. By (B1) and (B2), (*x*∗0)∗(*y*∗*y*) = *x*. Therefore *x* ∈ *S*, and thus *S* is an ideal. The converse follows from Proposition 17.

**Remark 22 .**

Let *I* be a normal ideal of a BN-algebra *𝒜*. For any *x*, *y* ∈ *A*, we define

(6) $$\begin{array}{c} x\;\sim_{I}\;y⟺x*y\in I. \end{array}$$

Then ~_*I*_ is a congruence of *𝒜* by the proof of Theorem 3.5 of [13].

**Definition 23 .**

Let *θ* be a congruence of a BN-algebra *𝒜*. One says that *θ* is* normal* if

(7) x∗yθ0, a∗bθ0 imply  x∗a∗y∗bθ0

for arbitrary *x*, *y*, *a*, *b* ∈ *A*.

**Example 24 .**

Let *A* = {0,1, 2,3} and ∗ be defined by the following:

(8) ∗012300123110322230133210

It is easy to check that *𝒜* = (*A*; ∗, 0) is a BN-algebra and *θ* = {(0,0), (1,1), (2,2), (3,3), (1,0), (0,1), (2,3), (3,2)} is a normal congruence of *𝒜*.

By *C*_*N*_(*𝒜*) we denote the set of all normal congruences of *𝒜*.

**Remark 25 .**

Let *𝒜* be a BN-algebra. It is easily seen that ~_*I*_ ∈ *C*_*N*_(*𝒜*), where *I* is a normal ideal of *𝒜*. In particular, *A* ^2^ = ~_*A*_ ∈ *C*_*N*_(*𝒜*).

**Proposition 26 .**

In BM-algebras all congruences are normal.

**Proof**

Let *𝒜* be a BM-algebra and let *θ* be a congruence of *𝒜*. Let *x*, *y*, *a*, *b* ∈ *A* and suppose that (*x*∗*y*)*θ*0 and (*a*∗*b*)*θ*0. Hence, ((*x*∗*y*)∗(*a*∗*b*))*θ*0. Observe that

(9) $$\begin{array}{c} \left(x*y\right)*\left(a*b\right)=\left(x*a\right)*\left(y*b\right). \end{array}$$

Indeed, we have

(10) x∗y∗a∗b=0∗y∗x∗a∗b by Proposition  2i=0∗a∗b∗y∗x by Proposition  2ii=b∗a∗y∗x by Proposition  2i=y∗a∗y∗b∗y∗x by BM=y∗a∗y∗x∗y∗b by Proposition  2ii=x∗a∗y∗b by BM.

Therefore, ((*x*∗*a*)∗(*y*∗*b*))*θ*0 and thus *θ* is normal.

**Remark 27 .**

Let *𝒜* = (*A*; ∗, 0) be the BN-algebra from Example 16. Observe that the least congruence on *𝒜*, namely, the identity relation *ω* = {(*a*, *a*) : *a* ∈ *A*}, is not normal. Indeed, we get (1∗3,0) ∈ *ω* and (2∗2,0) ∈ *ω* but ((1∗2)∗(3∗2), 0) = (2,0) ∉ *ω*.

Let *𝒜* be a BN-algebra and let *θ* be a congruence on *𝒜*. For *x* ∈ *A*, by [*x*]_*θ*_ we denote the congruence class containing *x*; that is, [*x*]_*θ*_ = {*y* ∈ *A* : *xθy*}.

**Proposition 28 .**

Let *θ* be a congruence on *𝒜*. Then *θ* ∈ *C*_*N*_(*𝒜*) if and only if [0]_*θ*_ is a normal ideal of *𝒜*.

**Proof**

Set *I* = [0]_*θ*_ and let *θ* ∈ *C*_*N*_(*𝒜*). It follows easily that 0 ∈ *I*. Let *x* and *y* be elements of *A* such that *x*∗*y*, *y* ∈ *I*. Then, (*x*∗*y*)*θ*0 and *yθ*0. Since *yθ*0, we obtain (*x*∗*y*)*θ*(*x*∗0) and hence *x* = *x*∗0 ∈ *I*. Consequently, *I* is an ideal. Now, suppose that *x*∗*y*, *a*∗*b* ∈ *I*, where *x*, *y*, *a*, *b* ∈ *A*. Then, (*x*∗*y*)*θ*0 and (*a*∗*b*)*θ*0. By the definition of a normal congruence, ((*x*∗*a*)∗(*y*∗*b*))*θ*0; that is, (*x*∗*a*)∗(*y*∗*b*) ∈ *I*. Thus, *I* = [0]_*θ*_ is normal.

The converse is obvious.

**Theorem 29 .**

There is a bijection between the set of normal ideals and the set of normal congruences of a BN-algebra.

**Proof**

Let *𝒜* be a BN-algebra. We consider functions *f* : Id_*N*_(*𝒜*) → *C*_*N*_(*𝒜*) and *g* : *C*_*N*_(*𝒜*) → Id_*N*_(*𝒜*) given as follows:

(11) fI~Ifor any  I∈IdNA,gθ=0θfor any  θ∈CNA.

Since ~_*I*_ ∈ *C*_*N*_(*𝒜*) and [0]_*θ*_ ∈ Id_*N*_(*𝒜*), we conclude that *f* and *g* are well-defined. Next, note that *I* = [0]_~_*I*__. Indeed,

(12) $$\begin{array}{c} x\in I⟺x*0\in I⟺x\;\sim_{I}\;0⟺x\in {\left[0\right]}_{\sim_{I}}. \end{array}$$

Hence,

(13) $$\begin{array}{c} \left(g\circ f\right)\left(I\right)=g\left(\sim_{I}\right)={\left[0\right]}_{\sim_{I}}=I\;\forall I\in \text{I}{\text{d}}_{N}\left(A\right). \end{array}$$

Further, observe that

(14) $$\begin{array}{c} x\theta y⟺\left(x*y\right)\theta 0⟺x*y\in {\left[0\right]}_{\theta } \end{array}$$

and from this we obtain

(15) $$\begin{array}{c} \left(f\circ g\right)\left(\theta \right)=f\left({\left[0\right]}_{\theta }\right)=\theta \;\forall \theta \in C_{N}\left(A\right). \end{array}$$

We deduce from 13 and 15 that *g*∘*f* = id_Id_*N*_(*𝒜*)_ and *f*∘*g* = id_*C*_*N*_(*𝒜*)_. Thus, *f* and *g* are inverse bijections between Id_*N*_(*𝒜*) and *C*_*N*_(*𝒜*).

Let *𝒜* = (*A*, ∗, 0_*A*_) and *ℬ* = (*B*, ∗, 0_*B*_) be BN-algebras. A mapping *f* : *A* → *B* is called a* homomorphism* from *𝒜* into *ℬ* if *f*(*x*∗*y*) = *f*(*x*)∗*f*(*y*) for any *x*, *y* ∈ *A*.

Observe that *f*(0_*A*_) = 0_*B*_. Indeed, *f*(0_*A*_) = *f*(0_*A*_∗0_*A*_) = *f*(0_*A*_)∗*f*(0_*A*_) = 0_*B*_. We denote by *ker*⁡*f* the subset {*x* ∈ *A* : *f*(*x*) = 0_*B*_} of *A* (it is the* kernel* of the homomorphism *f*).

**Lemma 30 .**

Let *f* : *A* → *B* be a homomorphism from *𝒜* into *ℬ*. Then *ker*⁡*f* is an ideal of *𝒜*.

**Proof**

Obviously, 0_*A*_ ∈ *ker*⁡*f*; that is, (I_1_) holds. Let *x*∗*y* ∈ *ker*⁡*f* and *y* ∈ *ker*⁡*f*. Then 0_*B*_ = *f*(*x*∗*y*) = *f*(*x*)∗*f*(*y*) = *f*(*x*)∗0_*B*_ = *f*(*x*). Consequently, *x* ∈ *ker*⁡*f*. Therefore, (I_2_) is satisfied. Thus *I* is an ideal of *𝒜*.

**Remark 31 .**

The kernel of a homomorphism is not always a normal ideal. Let (*A*; ∗, 0) be the algebra given in Example 16. Clearly, id_*A*_ : *A* → *A* is a homomorphism and the ideal *ker*⁡(id_*A*_) = {0} is not normal.

**Proposition 32 .**

Let *𝒜* = (*A*, ∗, 0_*A*_) be a BN-algebra and let *ℬ* = (*B*, ∗, 0_*B*_) be a BM-algebra. Let *f* : *A* → *B* be a homomorphism from *𝒜* into *ℬ*. Then *ker*⁡*f* is a normal ideal.

**Proof**

By Lemma 30, *ker*⁡*f* is an ideal of *𝒜*. Let *x*, *y*, *a*, *b* ∈ *A* and *x*∗*y*, *a*∗*b* ∈ *ker*⁡*f*. Then 0_*B*_ = *f*(*x*∗*y*) = *f*(*x*)∗*f*(*y*). From Proposition 3(ii) it follows that *f*(*x*) = *f*(*y*). Similarly, *f*(*a*) = *f*(*b*). Consequently, *f*((*x*∗*a*)∗(*y*∗*b*)) = (*f*(*x*)∗*f*(*a*))∗(*f*(*y*)∗*f*(*b*)) = (*f*(*x*)∗*f*(*a*))∗(*f*(*x*)∗*f*(*a*)) = 0_*B*_, and hence, (*x*∗*a*)∗(*y*∗*b*) ∈ *ker*⁡*f*.

Let *I* be a normal ideal of *𝒜*. For *x* ∈ *A*, we write *x*/*I* = {*y* ∈ *A* : *x*~_*I*_ *y*}; that is, *x*/*I* = [*x*]_~_*I*__. We note that

(16) $$\begin{array}{c} x\;\sim_{I}\;y⟺\frac{x}{I}=\frac{y}{I}. \end{array}$$

Denote *A*/*I* = {*x*/*I* : *x* ∈ *A*} and set *x*/*I*∗′*y*/*I* = *x*∗*y*/*I*. The operation ∗′ is well-defined, since ~_*I*_ is a congruence of *𝒜*. It is easy to see that *𝒜*/*I* = (*A*/*I*, ∗′, 0/*I*) is a BN-algebra. The algebra *𝒜*/*I* is called the* quotient BN-algebra of 𝒜 modulo I*.

**Example 33 .**

Let *𝒜* and *θ* be given as in Example 24. We know that *𝒜* is a BN-algebra and *θ* is a normal congruence of *𝒜*. Since [0]_*θ*_ = {0,1}, from Proposition 28 we see that *I* = {0,1} is a normal ideal of *𝒜*. We have 0/*I* = 1/*I* = {0,1} and 2/*I* = 3/*I* = {2,3}. Then *A*/*I* = {0/*I*, 2/*I*}. Clearly, 0/*I*∗′0/*I* = 0/*I* = 2/*I*∗′2/*I* and 0/*I*∗′2/*I* = 2/*I* = 2/*I*∗′0/*I*.

**Theorem 34 .**

Let *𝒜* be a BN-algebra and let *ℬ* be a BM-algebra. Let *f* : *A* → *B* be a homomorphism from *𝒜* onto *ℬ*. Then *𝒜*/*ker*⁡*f* is isomorphic to *ℬ*.

**Proof**

By Proposition 32, *I* = *ker*⁡*f* is a normal ideal of *𝒜*. Define a mapping *g* : *A*/*I* → *B* by *g*(*x*/*I*) = *f*(*x*) for all *x* ∈ *I*. Let *x*/*I* = *y*/*I*. Then, *x*~_*I*_ *y*; that is, *x*∗*y* ∈ *I*. Hence, *f*(*x*)∗*f*(*y*) = 0_*B*_. By Proposition 3(ii) we have *f*(*x*) = *f*(*y*). Consequently, *g*(*x*/*I*) = *g*(*y*/*I*). This means that *g* is well-defined. It is easy to check that *g* is a homomorphism from *𝒜*/*I* onto *ℬ*. Observe that *g* is one-to-one. Let *g*(*x*/*I*) = *g*(*y*/*I*). Then *f*(*x*) = *f*(*y*) and hence *f*(*x*∗*y*) = 0_*B*_; that is, *x*∗*y* ∈ *I*. Therefore, *x*~_*I*_ *y* and consequently, *x*/*I* = *y*/*I*. Thus *g* is an isomorphism from *𝒜*/*I* onto *ℬ*.

## 3. Fuzzy Ideals

We now review some fuzzy logic concepts. First, for Γ⊆[0; 1] we define ⋀Γ = inf⁡Γ and ⋁Γ = sup⁡Γ. Obviously, if Γ = {*α*, *β*}, then *α*∧*β* = min⁡{*α*, *β*} and *α*∨*β* = max⁡{*α*, *β*}. Recall that a* fuzzy set* in *A* is a function *μ* : *A* → [0; 1].

For any fuzzy sets *μ* and *ν* in *A*, we define

(17) $$\begin{array}{c} \mu \leq \nu ⟺\mu \left(x\right)\leq \nu \left(x\right)\;\forall x\in A. \end{array}$$

A trivial verification shows that this relation is an order relation in the set of fuzzy sets in *A*.

Let *A* and *B* be any two sets, *μ* any fuzzy set in *A*, and *f* : *A* → *B* any function. Set *f* ^←^(*y*) = {*x* ∈ *A* : *f*(*x*) = *y*} for *y* ∈ *B*. The fuzzy set *ν* in *B* defined by

(18) $$\begin{array}{c} \nu \left(y\right)=\left\{\begin{array}{cc} ⋁\left\{\mu \left(x\right):x\in f^{\leftarrow }\left(y\right)\right\}, & \text{if}\;\;f^{\leftarrow }\left(y\right)\neq ⌀,\\ 0, & \text{otherwise}, \end{array}\right. \end{array}$$

for all *y* ∈ *B*, is called the* image* of *μ* under *f* and is denoted by *f*(*μ*).

Let *A* and *B* be any two sets, *f* : *A* → *B* any function, and *ν* any fuzzy set in *f*(*A*). The fuzzy set *μ* in *A* defined by

(19) $$\begin{array}{c} \mu \left(x\right)=\nu \left(f\left(x\right)\right),\;\forall x\in A \end{array}$$

is called the* preimage* of *ν* under *f* and is denoted by *f* ^←^(*ν*).

Now, we give the definition of a fuzzy ideal in a BN-algebra.

**Definition 35 .**

A fuzzy set *μ* in *A* is called a* fuzzy ideal* of a BN-algebra *𝒜* if it satisfies, for all *x*, *y* ∈ *A*,

(d1) *μ*(0) ≥ *μ*(*x*),
(d2) *μ*(*x*) ≥ *μ*(*x*∗*y*)∧*μ*(*y*).

**Proposition 36 .**

Let *μ* be a fuzzy ideal of *𝒜*. Then, for any *x*, *y* ∈ *A*, if *x* ≤ *y*, then *μ*(*x*) ≤ *μ*(*y*).

**Proof**

If *x* ≤ *y*, then *x*∗*y* = 0. From Proposition 8(iv) we obtain *y*∗*x* = 0. Hence, by (d2) and (d1), we have *μ*(*y*) ≥ *μ*(*y*∗*x*)∧*μ*(*x*) = *μ*(0)∧*μ*(*x*) = *μ*(*x*).

Denote by *ℱ*Id(*𝒜*) the set of all fuzzy ideals of a BN-algebra *𝒜*.

**Example 37 .**

Let *𝒜* = (*A*, ∗, 0) be the BN-algebra given in Example 6. Let 0 ≤ *α*_3_ < *α*_2_ < *α*_1_ ≤ 1. Define a fuzzy set *μ* in *A* by

(20) $$\begin{array}{c} \mu \left(x\right)=\left\{\begin{array}{cc} \alpha_{1}, & \text{if}\;\;x=0,\\ \alpha_{2}, & \text{if}\;\;x=2,\\ \alpha_{3}, & \text{if}\;\;x\in \left\{1,3\right\}. \end{array}\right. \end{array}$$

It is easily checked that *μ* satisfies (d1) and (d2). Thus *μ* ∈ *ℱ*Id(*𝒜*).

**Example 38 .**

Let *I* be an ideal of a BN-algebra *𝒜* and let *α*, *β* ∈ [0; 1] with *α* ≥ *β*. Define *μ*_*I*_^*α*,*β*^ as follows:

(21) $$\begin{array}{c} \mu_{I}^{\alpha ,\beta }\left(x\right)=\left\{\begin{array}{cc} \alpha , & \text{if}\;\;x\in I,\\ \beta , & \text{otherwise}. \end{array}\right. \end{array}$$

We denote *μ*_*I*_^*α*,*β*^ = *μ*. Since 0 ∈ *I*, *μ*(0) = *α* ≥ *μ*(*x*) for all *x* ∈ *A*. To prove (d2), let *x*, *y* ∈ *A*. If *x* ∈ *I*, then *μ*(*x*) = *α* ≥ *μ*(*x*∗*y*)∧*μ*(*y*). Suppose now that *x* ∉ *I*. By the definition of an ideal, *x*∗*y* ∉ *I* or *y* ∉ *I*. Therefore, *μ*(*x*∗*y*)∧*μ*(*y*) = *β* = *μ*(*x*). Thus *μ* is a fuzzy ideal of *𝒜*.

In particular, the characteristic function *χ*_*I*_ of *I*:

(22) $$\begin{array}{c} \chi_{I}\left(x\right)=\left\{\begin{array}{cc} 1, & \text{if}\;\;x\in I,\\ 0, & \text{otherwise}, \end{array}\right. \end{array}$$

is a fuzzy ideal of *𝒜*.

**Proposition 39 .**

A fuzzy set *μ* in *A* is a fuzzy ideal of *𝒜* if and only if it satisfies (d1) and

(d3) for all *x*, *y*, *z* ∈ *A*, if (*z*∗*y*)∗*x* = 0, then *μ*(*z*) ≥ *μ*(*x*)∧*μ*(*y*).

**Proof**

Let *μ* ∈ *ℱ*Id(*𝒜*) and let *x*, *y*, *z* ∈ *A*. Suppose that (*z*∗*y*)∗*x* = 0. Since *μ* is a fuzzy ideal, we have *μ*(*z*∗*y*) ≥ *μ*((*z*∗*y*)∗*x*)∧*μ*(*x*) = *μ*(0)∧*μ*(*x*) = *μ*(*x*) and *μ*(*z*) ≥ *μ*(*z*∗*y*)∧*μ*(*y*). Therefore, *μ*(*z*) ≥ *μ*(*x*)∧*μ*(*y*).

Conversely, let *μ* satisfy (d3). From (B1) we have (*x*∗*y*)∗(*x*∗*y*) = 0. By (d3), *μ*(*x*) ≥ *μ*(*x*∗*y*)∧*μ*(*y*). Then *μ* satisfies (d2) and hence *μ* ∈ *ℱ*Id(*𝒜*).

**Theorem 40 .**

Let *μ* be a fuzzy set in *A*. Then *μ* ∈ *ℱ* Id (*𝒜*) if and only if its nonempty level subset

(23) $$\begin{array}{c} U\left(\mu ;\alpha \right)=\left\{x\in A:\mu \left(x\right)\geq \alpha \right\} \end{array}$$

is an ideal of *𝒜* for all *α* ∈ [0; 1].

**Proof**

Assume that *μ* ∈ *ℱ*Id(*𝒜*). Let *α* ∈ [0; 1] and *U*(*μ*; *α*) ≠ *∅*. Then *μ*(*x*_0_) ≥ *α* for some *x*_0_ ∈ *A*. Since *μ*(0) ≥ *μ*(*x*_0_), we have 0 ∈ *U*(*μ*; *α*). Let *x*, *y* ∈ *A* such that *x*∗*y*, *y* ∈ *U*(*μ*; *α*). Then *μ*(*x*∗*y*) ≥ *α* and *μ*(*y*) ≥ *α*. It follows from (d2) that

(24) $$\begin{array}{c} \mu \left(x\right)\geq \mu \left(x*y\right)\wedge \mu \left(y\right)\geq \alpha \end{array}$$

so that *x* ∈ *U*(*μ*; *α*). Therefore *U*(*μ*; *α*) is an ideal of *𝒜*.

Conversely, suppose that, for each *α* ∈ [0; 1], *U*(*μ*; *α*) = *∅* or *U*(*μ*; *α*) is an ideal of *𝒜*. If (d1) is not valid, then there exists *x*_0_ ∈ *A* such that *μ*(0) < *μ*(*x*_0_) = *β*. Then *U*(*μ*; *β*) ≠ *∅* and, by assumption, *U*(*μ*; *β*) is an ideal of *𝒜*. Hence 0 ∈ *U*(*μ*; *β*) and consequently *μ*(0) ≥ *β*. This is a contradiction and (d1) is valid. Now assume that (d2) does not hold. Then there are *a*, *b* ∈ *A* such that *μ*(*a*) < *μ*(*a*∗*b*)∧*μ*(*b*). Taking

(25) $$\begin{array}{c} \gamma =\frac{1}{2}\left(\mu \left(a\right)+\mu \left(a*b\right)\wedge \mu \left(b\right)\right), \end{array}$$

we get *μ*(*a*) < *γ* < *μ*(*a*∗*b*)∧*μ*(*b*) ≤ *μ*(*a*∗*b*) and *γ* < *μ*(*b*). Therefore *a*∗*b*, *b* ∈ *U*(*μ*; *γ*) but *a* ∉ *U*(*μ*; *γ*). This is impossible, and *μ* is a fuzzy ideal of *𝒜*.

By Theorem 40, we have the following.

**Corollary 41 .**

If *μ* is a fuzzy ideal of a BN-algebra *𝒜*, then the set

(26) $$\begin{array}{c} A_{\mu }=\left\{x\in A:\mu \left(x\right)=\mu \left(0\right)\right\} \end{array}$$

is an ideal of *𝒜*.

The following example shows that the converse of Corollary 41 does not hold.

**Example 42 .**

Let *𝒜* be a BN-algebra. Define a fuzzy set *μ* in *A* by

(27) $$\begin{array}{c} \mu \left(x\right)=\left\{\begin{array}{cc} 0.4, & \text{if}\;\;x=0,\\ 0.6, & \text{if}\;\;x\neq 0. \end{array}\right. \end{array}$$

Then *A*_*μ*_ = {0} is the ideal of *𝒜* but *μ* ∉ *ℱ*Id(*𝒜*) (because *μ* does not satisfy (d1)).

**Example 43 .**

Consider the BN-algebra *𝒜* = (*A*, ∗, 0) given in Example 6. Let *μ* be defined as in Example 37. It is easy to check that for all *α* ∈ [0; 1] we have

(28) $$\begin{array}{c} U\left(\mu ;\alpha \right)=\left\{\begin{array}{cc} \emptyset , & \text{if}\;\;\alpha >\alpha_{1},\\ \left\{0\right\}, & \text{if}\;\;\alpha_{2}<\alpha \leq \alpha_{1},\\ \left\{0,2\right\}, & \text{if}\;\;\alpha_{3}<\alpha \leq \alpha_{2},\\ A, & \text{if}\;\;\alpha \leq \alpha_{3}. \end{array}\right. \end{array}$$

Since {0}, {0,2} and *A* are ideals of *𝒜*, this is another proof (by Theorem 40) that *μ* is a fuzzy ideal of *𝒜*.

**Lemma 44 .**

Let *I*_1_ ⊂ *I*_2_ ⊂ ⋯⊂*I*_*n*_ ⊂ ⋯ be a strictly ascending sequence of ideals in a BN-algebra *𝒜* and let (*t*_*n*_) be a strictly decreasing sequence in (0; 1). Let *μ* be the fuzzy set in *A* defined by

(29) $$\begin{array}{c} \mu \left(x\right)=\left\{\begin{array}{cc} 0, & if\;\;x\notin I_{n}\;\;for\;\;each\;\;n\in N,\\ t_{n}, & if\;\;x\in I_{n}-I_{n-1}\;\;for\;\;some\;\;n\in N, \end{array}\right. \end{array}$$

where *I*_0_ = *∅*. Then *μ* is a fuzzy ideal of *𝒜*.

**Proof**

Let *I* = ⋃_*n*∈*ℕ*_ *I*_*n*_. By Remark 14, *I* is an ideal of *𝒜*. Obviously, *μ*(0) = *t*_1_ ≥ *μ*(*x*) for all *x* ∈ *A*; that is, (d1) holds. Now we show that *μ* satisfies (d2). Let *x*, *y* ∈ *A*. We have two cases.

*Case 1  *(*x* ∉ *I*). Then *x*∗*y* ∉ *I* or *y* ∉ *I*. Therefore *μ*(*x*∗*y*)∧*μ*(*y*) = 0 = *μ*(*x*).

*Case 2  *(*x* ∈ *I*_*n*_ − *I*_*n*−1_ for some *n* ∈ *ℕ*). Then *x*∗*y* ∉ *I*_*n*−1_ or *y* ∉ *I*_*n*−1_. Hence *μ*(*x*∗*y*) ≤ *t*_*n*_ or *μ*(*y*) ≤ *t*_*n*_. Therefore *μ*(*x*∗*y*)∧*μ*(*y*) ≤ *t*_*n*_ = *μ*(*x*).

Thus (d2) is also satisfied and consequently *μ* is a fuzzy ideal of *𝒜*.

Let *T* be a nonempty set of indexes. Let *μ*_*t*_ ∈ *ℱ*Id(*𝒜*) for *t* ∈ *T*. The meet ⋀_*t*∈*T*_ *μ*_*t*_ of fuzzy ideals *μ*_*t*_ of *𝒜* is defined as follows:

(30) $$\begin{array}{c} \left(\underset{t\in T}{⋀}\mu_{t}\right)\left(x\right)=⋀\left\{\mu_{t}\left(x\right):t\in T\right\}. \end{array}$$

**Theorem 45 .**

Let *μ*_*t*_ ∈ *ℱ* Id (*𝒜*) for *t* ∈ *T*. Then ⋀_*t*∈*T*_ *μ*_*t*_ ∈ *ℱ* Id (*𝒜*).

**Proof**

Let *μ* = ⋀_*t*∈*T*_ *μ*_*t*_. Then, by (d1),

(31) μ0⋀μt0:t∈T≥⋀μtx:t∈T=μx

for all *x* ∈ *A*. Let *x*, *y* ∈ *A*. Since *μ*_*t*_ ∈ *ℱ*Id(*𝒜*), we have *μ*_*t*_(*x*) ≥ *μ*_*t*_(*x*∗*y*)∧*μ*_*t*_(*y*). Hence

(32) ⋀μtx:t∈T⋀μtx∗y∧μty:t∈T=⋀μtx∗y:t∈T∧⋀μty:t∈T.

Consequently, *μ*(*x*) ≥ *μ*(*x*∗*y*)∧*μ*(*y*) and therefore *μ* ∈ *ℱ*Id(*𝒜*).

Let *ν* be a fuzzy set in *A*. A fuzzy ideal *μ* of *𝒜* is said to be* generated* by *ν* if *ν* ≤ *μ* and, for any fuzzy ideal *ρ* of *𝒜*, *ν* ≤ *ρ* implies *μ* ≤ *ρ*. The fuzzy ideal generated by *ν* will be denoted by (*ν*]. The fuzzy ideal (*ν*] can be defined equivalently as follows:

(33) $$\begin{array}{c} \left(\nu \right]=⋀\left\{\rho \in F\text{Id}\left(A\right):\rho \geq \nu \right\}. \end{array}$$

For *μ*, *ν* ∈ *ℱ*Id(*𝒜*) let *μ*∨*ν* denote the join of *μ* and *ν*; that is, *μ*∨*ν* = (*ρ*], where *ρ* is the fuzzy set in *A* defined by *ρ*(*x*) = *μ*(*x*)∨*ν*(*x*) for all *x* ∈ *A*.

From Theorem 45 we obtain the following theorem.

**Theorem 46 .**

Let *𝒜* be a BN-algebra. Then (*ℱ* Id (*𝒜*); ∧, ∨) is a complete lattice.

The following two theorems give the homomorphic properties of fuzzy ideals.

**Theorem 47 .**

Let *𝒜* and *ℬ* be BN-algebras and let *f* : *A* → *B* be a homomorphism and *ν* ∈ *ℱ* Id (*ℬ*). Then *f* ^←^(*ν*) ∈ *ℱ* Id (*𝒜*).

**Proof**

Let *x* ∈ *A*. Since *f*(*x*) ∈ *B* and *ν* ∈ *ℱ*Id(*ℬ*), we have *ν*(0) ≥ *ν*(*f*(*x*)) = (*f* ^←^(*ν*))(*x*), but *ν*(0) = *ν*(*f*(0)) = (*f* ^←^(*ν*))(0). Thus we get (*f* ^←^(*ν*))(0)≥(*f* ^←^(*ν*))(*x*) for any *x* ∈ *A*; that is, *f* ^←^(*ν*) satisfies (d1).

Now let *x*, *y* ∈ *A*. Since *ν* ∈ *ℱ*Id(*ℬ*), we obtain

(34) νfxνfx∗fy∧νfy=νfx∗y∧νfy

and hence (*f* ^←^(*ν*))(*x*)≥(*f* ^←^(*ν*))(*x*∗*y*)∧(*f* ^←^(*ν*))(*y*). Consequently, *f* ^←^(*ν*) ∈ *ℱ*Id(*𝒜*).

**Lemma 48 .**

Let *𝒜* and *ℬ* be BN-algebras and let *f* : *A* → *B* be a homomorphism and *μ* ∈ *ℱ* Id (*𝒜*). Then, if *μ* is constant on *ker*⁡*f* = *f* ^←^(0), then *f* ^←^(*f*(*μ*)) = *μ*.

**Proof**

Let *x* ∈ *A* and *f*(*x*) = *y*. Hence

(35) f←fμxfμfx=fμy=⋁μa:a∈f←y.

For all *a* ∈ *f* ^←^(*y*), we have *f*(*x*) = *f*(*a*). Hence *f*(*a*∗*x*) = 0; that is, *a*∗*x* ∈ *ker*⁡*f*. Thus *μ*(*a*∗*x*) = *μ*(0). Therefore, *μ*(*a*) ≥ *μ*(*a*∗*x*)∧*μ*(*x*) = *μ*(0)∧*μ*(*x*) = *μ*(*x*). Similarly, *μ*(*x*) ≥ *μ*(*a*). Hence *μ*(*x*) = *μ*(*a*). Thus

(36) $$\begin{array}{c} \left(f^{\leftarrow }\left(f\left(\mu \right)\right)\right)\left(x\right)=⋁\left\{\mu \left(a\right):a\in f^{\leftarrow }\left(y\right)\right\}=\mu \left(x\right); \end{array}$$

that is, *f* ^←^(*f*(*μ*)) = *μ*.

**Theorem 49 .**

Let *𝒜* and *ℬ* be BN-algebras and let *f* : *A* → *B* be a surjective homomorphism and *μ* ∈ *ℱ* Id (*𝒜*) such that *A*_*μ*_⊇*ker*⁡*f*. Then *f*(*μ*) ∈ *ℱ* Id (*ℬ*).

**Proof**

Since *μ* is a fuzzy ideal of *𝒜* and 0 ∈ *f* ^←^(0), we have

(37) $$\begin{array}{c} \left(f\left(\mu \right)\right)\left(0\right)=⋁\left\{\mu \left(a\right):a\in f^{\leftarrow }\left(0\right)\right\}=\mu \left(0\right)\geq \mu \left(x\right) \end{array}$$

for any *x* ∈ *A*. Hence

(38) $$\begin{array}{c} \left(f\left(\mu \right)\right)\left(0\right)\geq ⋁\left\{\mu \left(x\right):x\in f^{\leftarrow }\left(y\right)\right\}=\left(f\left(\mu \right)\right)\left(y\right) \end{array}$$

for any *y* ∈ *B*. Thus *f*(*μ*) satisfies (d1). Suppose that

(39) $$\begin{array}{c} \left(f\left(\mu \right)\right)\left(x_{B}\right)<\left(f\left(\mu \right)\right)\left(x_{B}*y_{B}\right)\wedge \left(f\left(\mu \right)\right)\left(y_{B}\right) \end{array}$$

for some *x*_*B*_, *y*_*B*_ ∈ *B*. Since *f* is surjective, there are *x*_*A*_, *y*_*A*_ ∈ *A* such that *f*(*x*_*A*_) = *x*_*B*_ and *f*(*y*_*A*_) = *y*_*B*_. Hence

(40) fμfxAfμfxA∗yA∧fμfyA.

Therefore

(41) f←fμxAf←fμxA∗yA∧f←fμyA.

Since *A*_*μ*_⊇*ker*⁡*f*, *μ* is constant on *ker*⁡*f*. Then, by Lemma 48, we get

(42) $$\begin{array}{c} \mu \left(x_{A}\right)<\mu \left(x_{A}*y_{A}\right)\wedge \mu \left(y_{A}\right), \end{array}$$

which is a contradiction with the fact that *μ* is a fuzzy ideal. Thus, we obtain *f*(*μ*) ∈ *ℱ*Id(*ℬ*).

## 4. Fuzzy Characterizations of Noetherian and Artinian BN-Algebras

In this section we characterize Noetherian BN-algebras and Artinian BN-algebras using some fuzzy concepts, in particular, fuzzy ideals.

A BN-algebra *𝒜* is called* Noetherian* if for every ascending sequence *I*_1_⊆*I*_2_⊆⋯ of ideals of *𝒜* there exists *k* ∈ *ℕ* such that *I*_*n*_ = *I*_*k*_ for all *n* ≥ *k*. A BN-algebra *𝒜* is called* Artinian* if for every descending sequence *I*_1_⊇*I*_2_⊇⋯ of ideals of *𝒜* there exists *k* ∈ *ℕ* such that *I*_*n*_ = *I*_*k*_ for all *n* ≥ *k*.

**Theorem 50 .**

Let *𝒜* be a BN-algebra. The following statements are equivalent:

- *𝒜* is Noetherian,
- for each fuzzy ideal *μ* of *𝒜*, *Im*⁡(*μ*) = {*μ*(*x*) : *x* ∈ *A*} is a well-ordered set.

**Proof**

(a) ⇒ (b): Assume that *𝒜* is Noetherian and *μ* is a fuzzy ideal of *𝒜* such that *Im*⁡(*μ*) is not a well-ordered subset of [0; 1]. Then there exists a strictly decreasing sequence (*μ*(*x*_*n*_)), where *x*_*n*_ ∈ *A*. Let *t*_*n*_ = *μ*(*x*_*n*_) and *U*_*n*_ = *U*(*μ*; *t*_*n*_) = {*x* ∈ *A* : *μ*(*x*) ≥ *t*_*n*_}. Then, by Theorem 40, *U*_*n*_ is an ideal of *𝒜* for every *n* ∈ *ℕ*. So *U*_1_ ⊂ *U*_2_ ⊂ ⋯ is a strictly ascending sequence of ideals of *𝒜*. This is a contradiction with the assumption that *𝒜* is Noetherian. Therefore *Im*⁡(*μ*) is a well-ordered set for each fuzzy ideal *μ* of *𝒜*.

(b) ⇒ (a): Assume that (b) is true. Suppose that *𝒜* is not Noetherian. Then there exists a strictly ascending sequence *I*_1_ ⊂ *I*_2_ ⊂ ⋯⊂*I*_*n*_ ⊂ ⋯ of ideals of *𝒜*. Let *μ* be a fuzzy set in *A* such that

(43) $$\begin{array}{c} \mu \left(x\right)=\left\{\begin{array}{cc} 0, & \text{if}\;\;x\notin I_{n}\text{ for each }n\in N,\\ \frac{1}{n}, & \text{if}\;\;x\in I_{n}-I_{n-1}\text{ for some }n\in N, \end{array}\right. \end{array}$$

where *I*_0_ = *∅*. By Lemma 44, *μ* ∈ *ℱ*Id(*𝒜*), but *Im*⁡(*μ*) is not a well-ordered set, which is impossible. Therefore *𝒜* is Noetherian.

**Corollary 51 .**

Let *𝒜* be a BN-algebra. If, for every fuzzy ideal *μ* of *𝒜*, *Im*⁡(*μ*) is a finite set, then *𝒜* is Noetherian.

**Theorem 52 .**

Let *𝒜* be a BN-algebra and let *T* = {*t*_1_, *t*_2_,…} ∪ {0}, where (*t*_*n*_) is a strictly decreasing sequence in (0; 1). Then the following conditions are equivalent:

- *𝒜* is Noetherian,
- for each fuzzy ideal *μ* of *𝒜*, if *Im*⁡(*μ*)⊆*T*, then there exists *k* ∈ *ℕ* such that *Im*⁡(*μ*)⊆{*t*_1_, *t*_2_,…, *t*_*k*_} ∪ {0}.

**Proof**

(a) ⇒ (b): Assume that *𝒜* is Noetherian. Let *μ* be a fuzzy ideal of *𝒜* such that *Im*⁡(*μ*)⊆*T*. From Theorem 50 we know that *Im*⁡(*μ*) is a well-ordered subset of [0; 1]. Then, since 1 > *t*_1_ > *t*_2_ > ⋯>*t*_*n*_ > ⋯>0 and *Im*⁡(*μ*)⊆{*t*_1_, *t*_2_,…} ∪ {0}, there exists *k* ∈ *ℕ* such that *Im*⁡(*μ*)⊆{*t*_1_, *t*_2_,…, *t*_*k*_} ∪ {0}.

(b) ⇒ (a): Assume that (b) is true. Suppose that *𝒜* is not Noetherian. Then there exists a strictly ascending sequence *I*_1_ ⊂ *I*_2_ ⊂ ⋯ of ideals of *𝒜*. Define a fuzzy set *μ* in *A* by

(44) $$\begin{array}{c} \mu \left(x\right)=\left\{\begin{array}{cc} 0, & \text{if}\;\;x\notin I_{n}\text{ for each }n\in N,\\ t_{n}, & \text{if}\;\;x\in I_{n}-I_{n-1}\text{ for some }n\in N, \end{array}\right. \end{array}$$

where *I*_0_ = *∅*. By Lemma 44, *μ* is a fuzzy ideal of *𝒜*. This is a contradiction with our assumption. Thus *𝒜* is Noetherian.

**Theorem 53 .**

Let *𝒜* be a BN-algebra and let *T* = {*t*_1_, *t*_2_,…} ∪ {0,1}, where (*t*_*n*_) is a strictly increasing sequence in (0; 1). Then the following conditions are equivalent:

- *𝒜* is Artinian,
- for each fuzzy ideal *μ* of *𝒜*, if *Im*⁡(*μ*)⊆*T*, then there exists *k* ∈ *ℕ* such that *Im*⁡(*μ*)⊆{*t*_1_, *t*_2_,…, *t*_*k*_} ∪ {0,1}.

**Proof**

(a) ⇒ (b): Suppose that *t*_*i*_1__ < *t*_*i*_2__ < ⋯<*t*_*i*_*m*__ < ⋯ is a strictly increasing sequence of elements of *Im*⁡(*μ*). Let *U*_*m*_ = *U*(*μ*; *t*_*i*_*m*__) for *m* ∈ *ℕ*. It is immediately seen that *U*_1_⊃*U*_2_⊃⋯⊃*U*_*m*_⊃⋯ is a strictly descending sequence of ideals of *𝒜*. This contradicts the assumption that *𝒜* is Artinian.

(b) ⇒ (a): Assume that (b) is true. Suppose that *𝒜* is not Artinian. Then there exists a strictly descending sequence *I*_1_⊃*I*_2_⊃⋯⊃*I*_*n*_⊃⋯ of ideals of *𝒜*. Define a fuzzy set *μ* in *A* by

(45) $$\begin{array}{c} \mu \left(x\right)=\left\{\begin{array}{cc} 0, & \text{if}\;\;x\notin I_{1},\\ t_{n}, & \text{if}\;\;x\in I_{n}-I_{n+1}\text{ for }n=1,2,\ldots ,\\ 1, & \text{if}\;\;x\in ⋂\left\{I_{n}:n\in N\right\}. \end{array}\right. \end{array}$$

Obviously, *μ*(0) = 1 ≥ *μ*(*x*) for all *x* ∈ *A*; that is, (d1) holds. Now we show that *μ* satisfies (d2). Let *x*, *y* ∈ *A*. We have three cases.

*Case 1  *(*x* ∉ *I*_1_). Then *x*∗*y* ∉ *I*_1_ or *y* ∉ *I*_1_. Therefore *μ*(*x*∗*y*)∧*μ*(*y*) = 0 = *μ*(*x*).

*Case 2  *(*x* ∈ *I*_*n*_ − *I*_*n*+1_ for some *n* ∈ *ℕ*). Then *x*∗*y* ∉ *I*_*n*+1_ or *y* ∉ *I*_*n*+1_. Hence *μ*(*x*∗*y*) ≤ *t*_*n*_ or *μ*(*y*) ≤ *t*_*n*_. Therefore *μ*(*x*∗*y*)∧*μ*(*y*) ≤ *t*_*n*_ = *μ*(*x*).

*Case 3  *(*x* ∈ ⋂{*I*_*n*_ : *n* ∈ *ℕ*}). It is obvious.

Thus *μ* is a fuzzy ideal of *𝒜*. This contradicts our assumption. Thus *𝒜* is Artinian.

**Corollary 54 .**

Let *𝒜* be a BN-algebra. If, for every fuzzy ideal *μ* of *𝒜*, *Im*⁡(*μ*) is a finite set, then *𝒜* is Artinian.

The following example shows that the converse of Corollary 54 does not hold.

**Example 55 .**

Let *p* be a prime number. Set *A* = {*z* ∈ *ℂ* : *z* ^*p*^*n*^^ = 1 for some *n* ≥ 0}. It is known that (*A*; ·, 1) is the *p*-quasicyclic group. Define *x*∗*y* = *xy* ^−1^ for all *x*, *y* ∈ *A*. By Example 7, *𝒜* = (*A*; ∗, 1) is a BN-algebra. Let *I*_*n*_ = {*z* ∈ *ℂ* : *z* ^*p*^*n*^^ = 1} for *n* ∈ *ℕ* ∪ {0}. It follows easily that *I* is an ideal of *𝒜* if and only if *I* = *A* or *I* = *I*_*n*_ for some *n* ≥ 0. We have *I*_0_ = {1} ⊂ *I*_1_ ⊂ ⋯⊂*I*_*n*_ ⊂ ⋯⊂*A* and hence *𝒜* is Artinian. Define *μ* by

(46) μx=1n+1if  x∈In−In−1 for  some  n∈N∪0,

where *I*_−1_ = *∅*. Since *A* = ⋃_*n*∈*ℕ*_ *I*_*n*_, *μ* is a fuzzy set in *A*. By the proof of Lemma 44, *μ* ∈ *ℱ*Id(*𝒜*). However, *Im*⁡(*μ*) = {1/*n* : *n* ∈ *ℕ*} is not a finite set.

## 5. Conclusions

This paper begins by considering the notion of ideals in BN-algebras. We give its characterizations and introduce the concept of normal ideals investigating its properties. We also define the notion of normal congruences proving that there is a one-to-one correspondence between normal ideals and normal congruences of a BN-algebra. Moreover we obtain the isomorphism theorem for BN-algebras. Next, we define the notion of fuzzy ideals of BN-algebras giving its characterizations and providing conditions for a fuzzy set to be a fuzzy ideal. We give the relationships between ideals and fuzzy ideals of a BN-algebra and also provide the homomorphic properties of fuzzy ideals. Finally, we display characterizations of Noetherian BN-algebras and Artinian BN-algebras via fuzzy ideals.

The next step in studying fuzzy ideals in BN-algebras may be introducing and investigating the notions of fuzzy maximal ideals and fuzzy prime ideals of BN-algebras.

## Figures and Tables

**Figure 1:**